# Measuring Criterion Validity of Microinteraction Ecological Momentary Assessment (Micro-EMA): Exploratory Pilot Study With Physical Activity Measurement

**DOI:** 10.2196/23391

**Published:** 2021-03-10

**Authors:** Aditya Ponnada, Binod Thapa-Chhetry, Justin Manjourides, Stephen Intille

**Affiliations:** 1 Khoury College of Computer Sciences Bouve College of Health Sciences Northeastern University Boston, MA United States; 2 Bouve College of Health Sciences Northeastern University Boston, MA United States

**Keywords:** ecological momentary assessment (EMA), experience sampling, physical activity, smartwatch, microinteractions, criterion validity, activity monitor, μEMA

## Abstract

**Background:**

Ecological momentary assessment (EMA) is an in situ method of gathering self-report on behaviors using mobile devices. In typical phone-based EMAs, participants are prompted repeatedly with multiple-choice questions, often causing participation burden. Alternatively, microinteraction EMA (micro-EMA or μEMA) is a type of EMA where all the self-report prompts are single-question surveys that can be answered using a 1-tap glanceable microinteraction conveniently on a smartwatch. Prior work suggests that μEMA may permit a substantially higher prompting rate than EMA, yielding higher response rates and lower participation burden. This is achieved by ensuring μEMA prompt questions are quick and cognitively simple to answer. However, the validity of participant responses from μEMA self-report has not yet been formally assessed.

**Objective:**

In this pilot study, we explored the criterion validity of μEMA self-report on a smartwatch, using physical activity (PA) assessment as an example behavior of interest.

**Methods:**

A total of 17 participants answered 72 μEMA prompts each day for 1 week using a custom-built μEMA smartwatch app. At each prompt, they self-reported whether they were doing sedentary, light/standing, moderate/walking, or vigorous activities by tapping on the smartwatch screen. Responses were compared with a research-grade activity monitor worn on the dominant ankle simultaneously (and continuously) measuring PA.

**Results:**

Participants had an 87.01% (5226/6006) μEMA completion rate and a 74.00% (5226/7062) compliance rate taking an average of only 5.4 (SD 1.5) seconds to answer a prompt. When comparing μEMA responses with the activity monitor, we observed significantly higher (*P*<.001) momentary PA levels on the activity monitor when participants self-reported engaging in moderate+vigorous activities compared with sedentary or light/standing activities. The same comparison did not yield any significant differences in momentary PA levels as recorded by the activity monitor when the μEMA responses were randomly generated (ie, simulating careless taps on the smartwatch).

**Conclusions:**

For PA measurement, high-frequency μEMA self-report could be used to capture information that appears consistent with that of a research-grade continuous sensor for sedentary, light, and moderate+vigorous activity, suggesting criterion validity. The preliminary results show that participants were not carelessly answering μEMA prompts by randomly tapping on the smartwatch but were reporting their true behavior at that moment. However, more research is needed to examine the criterion validity of μEMA when measuring vigorous activities.

## Introduction

Ecological momentary assessment (EMA), also known as the experience sampling method, is used to measure behaviors of people in natural settings [1]. In a typical EMA study, a user’s phone is prompted multiple times a day (often 6+ times) with a set of multiple-choice questions measuring behaviors of interest [2,3]. The repeated EMA prompts, which typically ask about momentary behaviors or states, not only reduce recall biases present in retrospective surveys [3,4] but also capture temporal changes in health behaviors unique to each individual [5]. Because of these benefits, EMA is commonly used to measure behaviors in intensive longitudinal studies [6].

The drawback of EMA is participation burden [7-9]. Participants are first interrupted with a beep and/or vibration. They must then find the phone, unlock the device, and respond to a set of complex multiple-choice questions. This repeated effort, which can take tens of seconds for even the shortest surveys and several minutes for many common surveys, can be burdensome, negatively impacting study compliance [7,9,10]. Microinteraction EMA (μEMA or micro-EMA) is a type of EMA that may, for some behaviors (eg, chronic pain or fatigue), enable high frequency self-report data collection with low study burden [11]. In μEMA, rather than using complex multiquestion surveys, each prompt contains only a single question that can be answered with a glanceable microinteraction [12], typically just a tap on a smartwatch. Prior studies have shown that despite approximately 8 times more interruption than EMA, μEMA had a significantly higher response rate and lower perceived burden because all interactions are limited to microinteractions [11,13]. Thus, there is preliminary evidence that μEMA may enable gathering high-frequency self-report with manageable burden, a complementary approach to EMA. Recently, μEMA has been used to gather data on stress [14], hyperarousal [15], and perceived comfort [16], and it has also been used with small pervasive displays [17].

Prior work on μEMA, however, has assumed validity of μEMA responses and not demonstrated it. Because μEMA is designed to gather small amounts of information with each prompt (but with higher frequency), the prompts are both limited to a single question and made cognitively simple to answer with a quick microinteraction (taking only 3-5 seconds). To achieve cognitive simplicity and fit questions on a smartwatch so they can be answered in a single tap without scrolling requires a limited answer set. This calls into question whether μEMA responses could capture behavior similarly to a gold-standard instrument (ie, criterion validity [18]), and validating such a high-frequency self-report requires an instrument that can measure the same behavior continuously in free living, such as a wearable sensor. In some domains where μEMA may be especially useful (eg, chronic pain), such sensors do not yet exist. The purpose of this pilot study is to explore the criterion validity of μEMA self-report, and thus, we used the example of physical activity (PA) measurement, because PA can be estimated continuously using research-grade activity monitors [19].

## Methods

In this pilot study, we compared μEMA self-report on a smartwatch with acceleration data collected using a wearable activity monitor on the dominant ankle to assess criterion validity, such as whether participants are answering the μEMA questions meaningfully in a way that changes as PA changes.

### μEMA App

**Figure 1:**
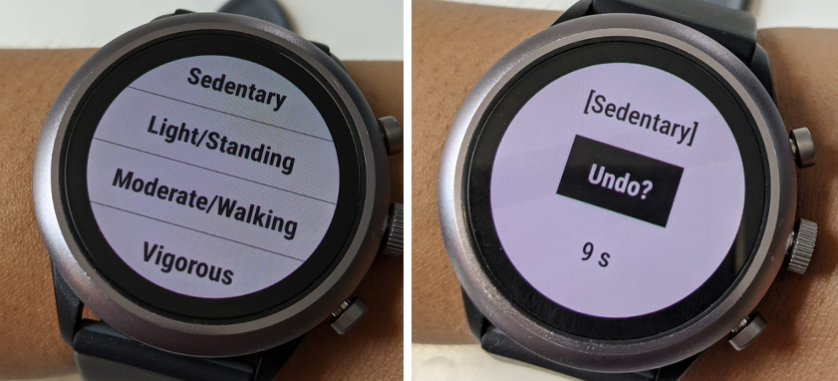
(Left) μEMA interface on a smartwatch with four activity intensity options. (Right) Undo screen to change response, available for 10 s, with a countdown timer.

We implemented a μEMA app on an Android Wear OS 2.0 (Figure 1) to measure PA. PA was chosen because (1) it can be estimated continuously using a passive, easy-to-wear sensor (eg, an accelerometer on the ankle) and (2) PA can change frequently within a day, making it suitable for testing a high-frequency μEMA self-report system. Participants were presented with 4 activity intensity options with each μEMA prompt: sedentary, light/standing, moderate/walking, and vigorous.

The μEMA app prompted 6 times an hour between 8 AM to 8 PM (72 expected prompts per day) using vibration on the smartwatch. The question displayed at the start of the vibration consisted of the 4 activity intensity categories, and participants selected the activity intensity they were engaged in at that moment. If the participant did not respond to the prompt within 2 minutes by tapping on a category, the prompt disappeared from the screen and the app recorded a missed response. When a response was selected, the watch displayed an Undo? screen (Figure 1). If participants tapped on the Undo? button, they were returned to the μEMA question to change their responses, otherwise the Undo? screen disappeared after 10 seconds. Data from the watch were sent to the participant’s smartphone once per hour. Participants interacted only with the watch, and the phone collected, encrypted, and transferred data from the watch to a remote server.

### Study Design

We conducted a week-long, within-subject pilot study (approved by Northeastern University’s institutional review board; project number 14-10-01) to compare μEMA PA responses with a wearable sensor.

#### Participant Recruitment

Participants were eligible if they owned a compatible Android smartphone with version 4.3+, were aged 18 to 55 years, were a student or staff at our university (to ensure we could safely recover loaned smartwatches and activity monitors at the end of the study), and were willing to wear a sensor and the smartwatch for 1 week. The study was advertised using flyers posted on the university campus and also by sending electronic notices to common university announcement portals. Of the 35 people who responded to study advertisements, 20 were eligible to participate based on screening via phone call. Among those, 3 participants dropped out early. One had a wake-period outside of μEMA prompting hours, another had a job that physically made it difficult to answer prompts on the watch, and the third had a malfunctioning phone. This left 17 active participants in the pilot study (11 males and 6 females; aged 19 to 34 years). None of the participants were affiliated to our research group.

#### Measurement Tasks

Participants were asked to complete 2 tasks simultaneously between 8 AM to 8 PM every day for one week: answer μEMA prompts on the loaned smartwatch (model Urbane, LG Electronics) and wear an activity monitor on their dominant ankle. Participants were asked to ignore μEMA prompts in unsafe conditions (eg, driving) and charge the watch nightly so that it could be worn next morning.

**Figure 2:**
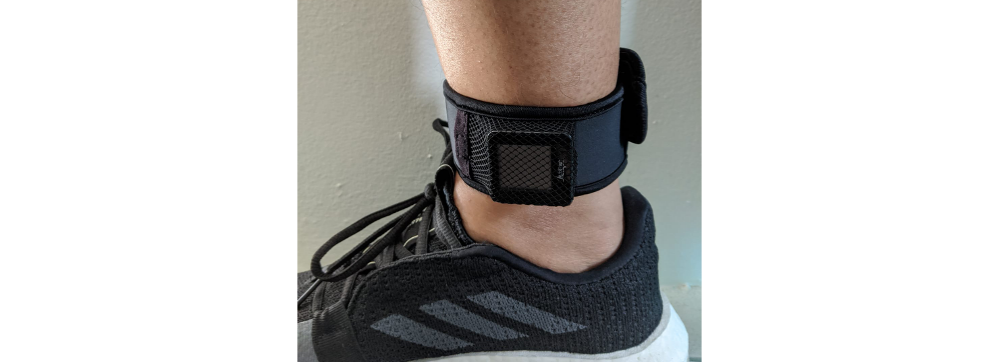
GT9X activity monitor worn on the dominant ankle.

We used GT9X monitors (35×35 mm, 14 g; ActiGraph LLC) to measure acceleration continuously at 80 Hz [20]. Participants wore the sensor on the dominant ankle above the medial malleolus using an elastic band (Figure 2). The ankle location was chosen because ankle acceleration can reliably capture ambulation activities, more so than the wrist or hip [21,22]. The sensor collected raw acceleration passively; other than wearing it, the participants did not interact with, or charge, this device.

#### Procedures

Day 0: Researchers met with participants, obtained informed consent, and loaned the activity monitor and smartwatch with the μEMA app. Research staff then presented participants with some examples of the types of different activities that would fall into each of the 4 target activity categories (Multimedia Appendix 1). Days 1-7: Participants wore the activity monitor on the dominant ankle and answered 6 μEMA prompts per hour between 8 AM to 8 PM on the smartwatch for 1 week. Day 8: Researchers recovered the monitor. Participants were not compensated financially to ensure that we measure criterion validity without any external motivation, but participants could use the smartwatch for 4 more days as their personal device for fun if they desired. All participants agreed to use the device for 4 more days.

## Results

We computed participants’ (1) compliance rate, the percentage of μEMA prompts answered out of all the scheduled prompts (ie, including when the watch was off); (2) completion rate, the percentage of μEMA prompts answered out of delivered prompts (ie, excluding when the watch was off); and (3) response time, the time taken to answer a prompt, measured from the start of the prompt vibration.

**Figure 3:**
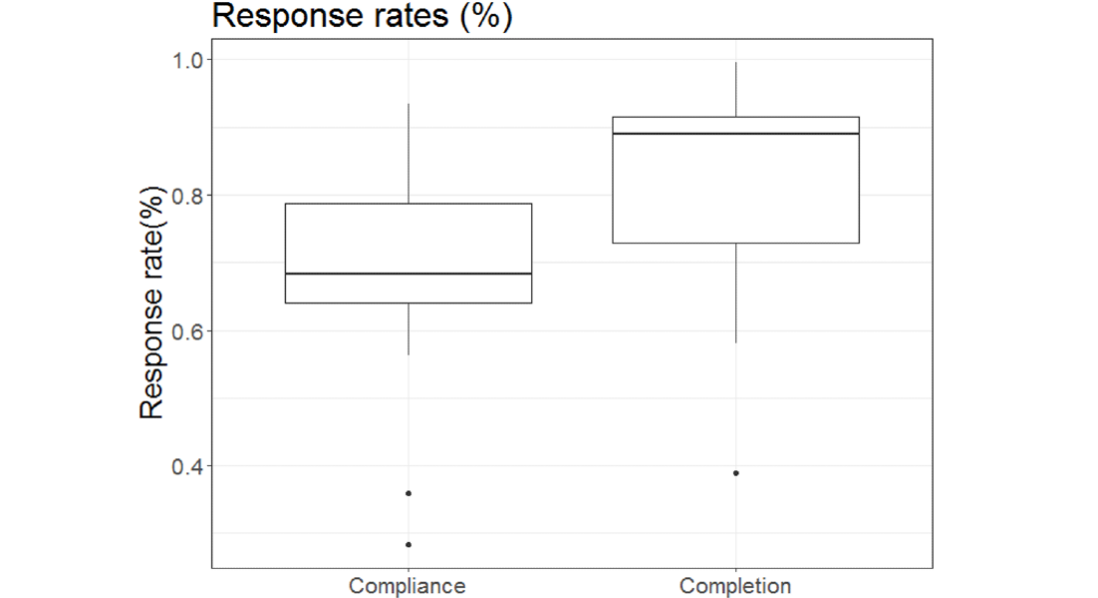
μEMA compliance and completion rates.

Two participants had low compliance (Figure 3). From the debriefing, we learned that these participants did not charge the smartwatch regularly, receiving fewer scheduled prompts. Their compliance fell below the 1.5 interquartile range (<40%); therefore, they were considered outliers and were excluded from the main analysis of data from the remaining 15 participants (Table 1) [23]. Implications of dropping the outliers is discussed later in our results.

**Table 1:** Response behavior of pilot study participants.

Characteristics	With outliers (n=17)	Without outliers (n=15)
Expected prompts, n	7591	7062
Delivered prompts, n	6387	6006
Answered prompts, n	5238	5226
Compliance rate (%)	69.00	74.00
Completion rate (%)	82.01	87.01
Response time (s), mean (SD)	5.5 (1.6)	5.4 (1.5)

### Data Preparation

#### Computing Activity Counts

ActiGraph activity counts are widely used motion summary metrics computed from raw acceleration for a specified epoch [24]. Activity counts have been used in prior work to compare EMA responses and accelerometer data [25,26]. We first computed activity counts for 1 second epochs of the raw data from the ankle-worn activity monitor. Using this, we calculated the total activity counts 60 seconds prior to the μEMA prompt as the PA level measured using the activity monitor.

#### Removing Sensor Nonwear Data

We removed the instances of raw data when the participants were not wearing the activity monitor. Following Choi et al [27], 90+ minutes of continuous zero-valued activity counts computed for the 1 second epochs were considered sensor nonwear times. All μEMA responses recorded during these nonwear times were dropped. This eliminated only 1.3% (70/5226) of the total responses from 15 participants leaving 5156 valid responses with sensor wear. Of these, 25% (18/70) of the responses were from just 1 participant.

#### μEMA Response Distribution

We received more sedentary responses (3619/5156, 69.99%) than light/standing (978/5156, 18.97%) and moderate/walking (544/5156, 10.56%) from μEMA, which is consistent with general physical (in)activity trends [28,29]. However, we received few vigorous responses (15/5156, 0.29%). Thus, we combined the moderate/walking and vigorous categories into a single category of moderate+vigorous. Hence, we compare the 3 PA intensities (sedentary, light/standing, and moderate+vigorous) from μEMA with activity count (60 seconds prior to the prompt) from the ankle-worn activity monitor (Table 2). These activity counts were within the ranges recorded previously in young adults for sedentary, light, and moderate+vigorous activities using ankle-worn accelerometers [30-33].

**Figure 4:**
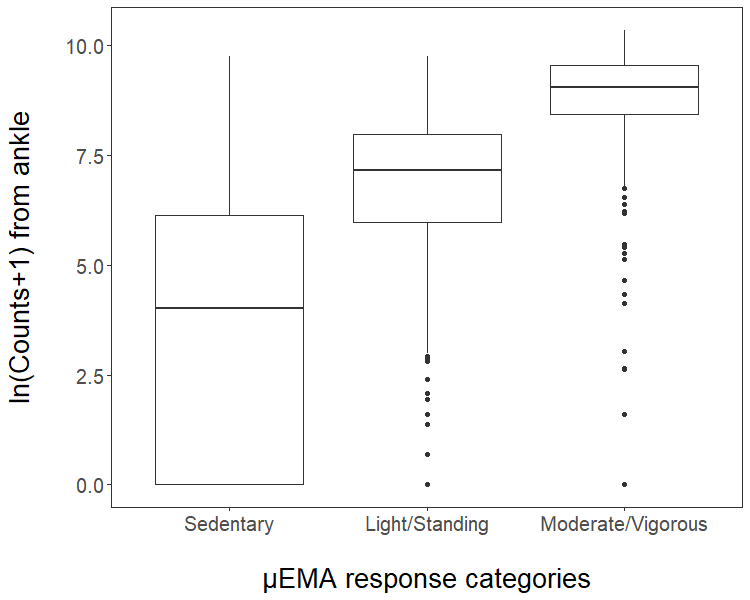
μEMA responses versus ln(Counts + 1).

Activity counts computed 60 seconds before the prompt ranged from 0 (for sedentary) to >15K (for moderate/vigorous), and resulted in a right-skewed distribution. Thus, we log-transformed these activity counts into ln(Counts + 1), where 1 is the smallest nonzero count recorded in this pilot study [34]. Figure 4 presents the final distribution of these log-transformed counts corresponding to μEMA categories (sedentary, light/standing, and moderate+vigorous).

**Table 2:** ln(Counts + 1) measured on ankle for each μEMA category.

Category		Value
**Sedentary**		
	Mean (SD)	3.56 (2.94)
	Median (IQR^a^)	4.01 (6.13)
**Light/standing**		
	Mean (SD)	6.66 (2.10)
	Median (IQR)	7.16 (2.01)
**Moderate+vigorous**		
	Mean (SD)	8.72 (1.52)
	Median (IQR)	9.04 (1.11)

^a^IQR: interquartile range.

### Criterion Validity of μEMA on Smartwatch

We applied a linear mixed-effects model with a random intercept (using the lme4 package [35]):

ln(*Counts_ij_* + 1) = 𝛽_0_ + 𝛽_1_ (*𝜇EMA_ij_*) + *u_i_*

Here, Counts_ij_ is the activity count from the ankle-worn monitor measured for an individual *i* computed for 60 seconds before μEMA prompt *j*, β_0_ is the fixed-effect intercept, μEMA_ij_ is the ordinal self-report (0 = sedentary, 1 = light/standing, and 2 = moderate/vigorous) on the smartwatch by participant *i* at prompt *j*, and u_i_ is the random-intercept for the participant *i*. Although we were interested in the fixed-effects part of the model, the random intercept is included to account for repeated measures within participants. The momentary PA levels measured on the activity monitor, ln(Counts_ij_ + 1), were significantly different (*P*<.001) for sedentary, light/standing, and moderate+vigorous activity categories captured using μEMA self-report (ie, μEMA_ij_). The final model fit for this pilot study was:

ln(*Counts_ij_* + 1) = 6.28 + 3.57 (*𝜇EMA_ij_*) + *u_i_*

We then included data from the two outliers to explore the sensitivity of the model fit and the activity levels, ln(Counts_ij_ + 1) were significantly different (*P*<.001) for the 3 μEMA response categories (μEMA_ij_). However, we observed that these participants showed continuous sensor nonwear for 2 to 3 days at once in addition to not charging the smartwatch regularly as required by the study protocol. In fact, removing the sensor nonwear for these participants eliminated 27.78% (30/108) and 43.04% (65/151) of the answered μEMA prompts, respectively. As a result, these participants were excluded from our final model fit. For the remaining 15 participants, the pair-wise, post hoc comparison of the 3 μEMA response categories with Tukey adjustment revealed that ln(Counts_ij_ + 1) for sedentary responses were significantly less than light/standing, which were significantly less than the moderate+vigorous (*P*<.001), the expected order of activity intensity (Table 3). In other words, when participants self-reported (using μEMA) being in moderate+vigorous instead of light/standing or sedentary activities, they were also more likely to have passively measured higher PA levels in those moments.

For exploratory purposes, we simulated random μEMA responses for each participant; instead of analyzing their actual responses, we compared the random data as if participants were randomly tapping on the watch screen only to dismiss the prompt with motion data recorded from the ankle. The model fit did not yield any significant differences in the counts for different μEMA categories. This further suggests that participants were answering μEMA questions carefully, not randomly (or carelessly), despite the intensive sampling rate.

**Table 3:** Pairwise comparison of ln(Counts + 1) from activity monitor with μEMA responses.

μEMA^a^ response (i)	μEMA response (j)	Mean difference (i–j)	SE	95% CI
Sedentary	Light/standing	–3.12	0.28	–3.34 to –2.90
Light/standing	Moderate+vigorous	–1.93	0.13	–2.24 to –1.16
Moderate+vigorous	Sedentary	5.05	0.11	4.78 to 5.32

^a^μEMA: microinteraction ecological momentary assessment.

## Discussion

### Principal Findings

In this pilot study, we explored criterion validity of μEMA on a smartwatch by measuring PA intensity of free-living individuals. For 1 week, 15 participants answered 6 μEMA prompts per hour (between 8 AM to 8 PM each day) reporting their PA intensity while simultaneously wearing an ankle-worn research-grade activity monitor. The activity monitor gathered raw accelerometer data continuously at 80 Hz. When comparing the μEMA self-report and activity monitor, we observed significantly higher momentary PA levels on the activity monitor when participants self-reported engaging in moderate+vigorous activities compared with when they reported sedentary or light/standing activities. Similarly, we observed significantly higher PA levels on the activity monitor when participants self-reported engaging in light/standing activities compared with when they reported being sedentary. Thus, we observed the expected order of intensity of these activity categories, suggesting criterion validity of μEMA self-report. Holistically, this result is aligned with the prior work where PA researchers have compared phone-based EMA with body-worn research-grade activity monitors measuring raw acceleration and found EMA to measure PA similar to the objective sensor [25,36,37]. However, the frequency of EMA self-report in these studies varied from once a day [25] to once an hour [37] to minimize interruption burden. In our pilot study, we extend these findings to an alternate self-report approach (μEMA) that allowed for 6 times more temporal density in measurement, yielding high response rate but without burdening participants as much as the phone based EMA.

Overall, this preliminary result shows that when measuring PA, participants’ μEMA self-report (at a high temporal density) could capture the PA levels consistent with a continuous high-frequency sensor. It appears, for example, that participants answered μEMA prompts meaningfully—not just tapping on an answer to dismiss the prompt—and sustained this answering at a rate of 6 times per hour for 12 hours per day for an entire week (approximately 504 prompts per person). Our preliminary findings suggest that the μEMA prompts achieve the goal of being so easy to answer that they can be sustained, instead of being ignored or dismissed—all while recording the true behavior at that moment. Participants know that every μEMA prompt just requires a single, 1-tap response on the easily accessible smartwatch that can be completed in a microinteraction; this may contribute to high compliance and valid data entry. EMA protocols, alternatively, often require answering multiple, sometimes complex, questions that can be time consuming and feel burdensome. In fact, the effort needed to dismiss an μEMA prompt is roughly equivalent to the effort required to answer the prompt, thus encouraging survey completion.

### Limitations

This exploratory pilot study provides preliminary findings on criterion validity of μEMA, but more research is needed. We merged the vigorous and moderate/walking activities into moderate+vigorous because we received only 15 responses of vigorous activities. One reason may be that most individuals engage in significantly more sedentary than vigorous activities [38]. Another may be that during the exit debriefing, 2 participants reported that when they engaged in vigorous activities like outdoor cycling, they could not respond to prompts within 2 minutes, thus resulting in fewer vigorous activity responses. This type of response behavior during vigorous activities has also been observed in a prior EMA versus activity monitor validation study where more missing responses were found during vigorous activity [37]. Nevertheless, this potential for a bias when reporting vigorous activity using μEMA should be explored in future work. If a reporting bias for vigorous activity is observed in future studies, one remedy could be to explore sensor-triggered μEMA, where the μEMA prompts might be presented based on real-time processing of PA and then delivered not during vigorous PA but rather right after it is confidently estimated to have been completed [39]. This also highlights the need to rethink question wording, where instead of asking about behavior in the moment (eg, Doing vigorous PA now?), μEMA will have to ask about the recent past (eg, Vigorous PA 2 min ago?) but without compromising the cognitive simplicity required for a microinteraction. Notably, EMA delivered via a smartphone would have likely been even more difficult to respond to during vigorous PA due to the difficulty of accessing and interacting with the phone device (versus the comparative simplicity of completing a microinteraction on the smartwatch) [40]. Future studies with larger sample sizes including individuals recruited specifically because they are known to regularly engage in vigorous activities could provide more insights on μEMA validity when measuring vigorous activity.

Being an exploratory pilot study, we had a small sample size. We were limited by available equipment, and recruiting was more challenging than in typical studies because we did not offer financial compensation for study participation (contrary to most phone EMA studies); our intent was to measure μEMA compliance and data quality in a situation without any external monetary incentives because such incentives may not be viable for future longitudinal measurement or intervention studies that might use μEMA.

Because validity assessments are domain-dependent, our findings with PA do not necessarily generalize to other behaviors. PA was chosen in this work because we have research-grade activity monitors to compare μEMA responses against at a high temporal density, not necessarily because PA intensity is best measured with μEMA if passive sensors are available. However, in some domains where μEMA might be useful (eg, chronic pain), passive sensors that continuously monitor the behavior are not available yet, and methods such as direct or physiological observations that require laboratory conditions are not practical for multiday free-living studies. Nevertheless, validation studies generally rely on imperfect comparisons, and so confidence in validity of μEMA, just like EMA, will require multiple cross-domain and longitudinal experiments from different research teams, of which this may be the first of many.

### Conclusion

The μEMA implemented on a smartwatch addresses the common Achilles’ heel of traditional phone-based EMA, the participation burden of accessing, unlocking, and answering multiple multichoice questions on a smartphone. Despite significantly higher interruption rates than phone-based EMA, μEMA is able to yield significantly higher response rates with manageable participation burden. This is achieved by keeping the single questions in μEMA cognitively simple to answer allowing for high frequency of prompting (like a continuous sensor). However, this makes μEMA vulnerable to careless tapping on the smartwatch to dismiss the prompts, potentially compromising the validity of self-report responses. Thus, in this pilot study, we explored the criterion validity of μEMA self-report, comparing it with a continuous sensor to assess if participants submit their responses based on their true behavior in the moment. We used PA as an example domain because PA can be measured using a gold-standard sensor (research-grade accelerometers). We conducted a 1-week exploratory pilot study with 15 participants answering 72 μEMA prompts each day while measuring continuous PA using an ankle-worn accelerometer. We found that participants were able to correctly report their sedentary, light/standing, and moderate+vigorous activities. This highlights that participants were not carelessly tapping on the smartwatch only to dismiss the prompt but were providing accurate information about their behavior comparable to the continuous sensor data from the ankle suggesting criterion validity. However, more research is needed to explore criterion validity of μEMA in other behavioral domains of interest including vigorous PA measurement.

## Appendix

Multimedia Appendix 1 Onboarding instructions.

## Abbreviations

EMA: ecological momentary assessment
PA: physical activity
μEMA: microinteraction ecological momentary assessment
