# Supplementary material for: Measuring Criterion Validity of Microinteraction Ecological Momentary Assessment (Micro-EMA): Exploratory Pilot Study With Physical Activity Measurement
Source: JMIR Mhealth Uhealth. 2021 Mar 10;9(3):e23391. doi: 10.2196/23391 (PMC7991987; doi:10.2196/23391)
Supplement: Multimedia Appendix 1 [file mhealth_v9i3e23391_app1.docx]

### Multimedia Appendix 1: Onboarding Instructions

Before beginning the study, participants were provided the following activity examples for each uEMA response category. The list was *only* to provide a reference of activity examples that correspond to different activity categories. Participants were told to choose the most relevant category of activity (i.e., μEMA response) given the activity they were engaged in when the vibration prompt started on the watch.

| **μEMA response categories** | **Examples provided to participants** |
| --- | --- |
| Sedentary | Sitting, lying down, sitting and eating, and similar activities |
| Light/Standing | Standing still, standing and eating, waiting for bus, and similar activities |
| Moderate/Walking | Walking, brisk walking, taking stairs, and similar activities |
| Vigorous | Running, jogging, cycling, and similar activities |
